# Accidental Genetic Engineers: Horizontal Sequence Transfer from Parasitoid Wasps to Their Lepidopteran Hosts

**DOI:** 10.1371/journal.pone.0109446

**Published:** 2014-10-08

**Authors:** Sean E. Schneider, James H. Thomas

**Affiliations:** Department of Genome Sciences, University of Washington, Seattle, Washington, United States of America; University of Poitiers, France

## Abstract

We show here that 105 regions in two Lepidoptera genomes appear to derive from horizontally transferred wasp DNA. We experimentally verified the presence of two of these sequences in a diverse set of silkworm (*Bombyx mori*) genomes. We hypothesize that these horizontal transfers are made possible by the unusual strategy many parasitoid wasps employ of injecting hosts with endosymbiotic polydnaviruses to minimize the host's defense response. Because these virus-like particles deliver wasp DNA to the cells of the host, there has been much interest in whether genetic information can be permanently transferred from the wasp to the host. Two transferred sequences code for a BEN domain, known to be associated with polydnaviruses and transcriptional regulation. These findings represent the first documented cases of horizontal transfer of genes between two organisms by a polydnavirus. This presents an interesting evolutionary paradigm in which host species can acquire new sequences from parasitoid wasps that attack them. Hymenoptera and Lepidoptera diverged ∼300 MYA, making this type of event a source of novel sequences for recipient species. Unlike many other cases of horizontal transfer between two eukaryote species, these sequence transfers can be explained without the need to invoke the sequences ‘hitchhiking’ on a third organism (e.g. retrovirus) capable of independent reproduction. The cellular machinery necessary for the transfer is contained entirely in the wasp genome. The work presented here is the first such discovery of what is likely to be a broader phenomenon among species affected by these wasps.

## Introduction

The transfer of genetic information between different species, horizontal gene transfer (HGT), is an unexpected and transformational discovery resulting from the expansion of whole genome sequence data. Examples of HGT violate the traditional idea that shared sequences among organisms come from a shared ancestral species. HGT can be a source of novel sequences that organisms would otherwise not have access to [1] such as a fungal carotenoid gene that gave pea aphids a bright orange coloration [2] or antifreeze genes transferred between very distant fish species [3]. Not all horizontal transfers are clearly adaptive. There are a number of cases where transposons have been transferred between parasite species and their host species [4], [5], [6], [7], [8].

Nevertheless, HGT is rarely reported among multicellular eukaryotes, presumably due to a combination of the poor efficiency of foreign sequences entering the germline and the difficulties of detecting HGT events. Viruses, however, can enter cells and facilitate HGT. Many fragments of virus genomes have been found in eukaryotes [9], and these endogenous viral elements provide a rich source of information on the evolution and history of viruses. Here we present horizontally transferred sequences (HTS) from polydnaviruses which were created by a process similar to the process by which endogenous viral elements are formed. A key difference between EVEs and polydnaviruses is that instead of transferring viral sequences, PDVs transfer genetic material from specific portions of the wasp genome.

Polydnaviruses (PDVs) are an unusual group of virus-like particles named after their dsDNA genomes that exist in many circular fragments [10]. PDVs are utilized alongside or instead of venom to aid in parasitoid reproduction by weakening the secondary host's immune system so that the parasitoid eggs can develop. Unlike typical viruses, PDVs are incapable of self-replication and instead are endosymbiotic viruses of two clades of parasitic wasps, Braconids (bracoviruses) and Ichneumonids (ichnoviruses). These two wasp clades have stably integrated viral sequences into their genomes and inherit them vertically [11], [12], [13], [14]. Viral particle production occurs exclusively in the ovaries of these parasitoid wasps [15], [16].

**Figure 1:**
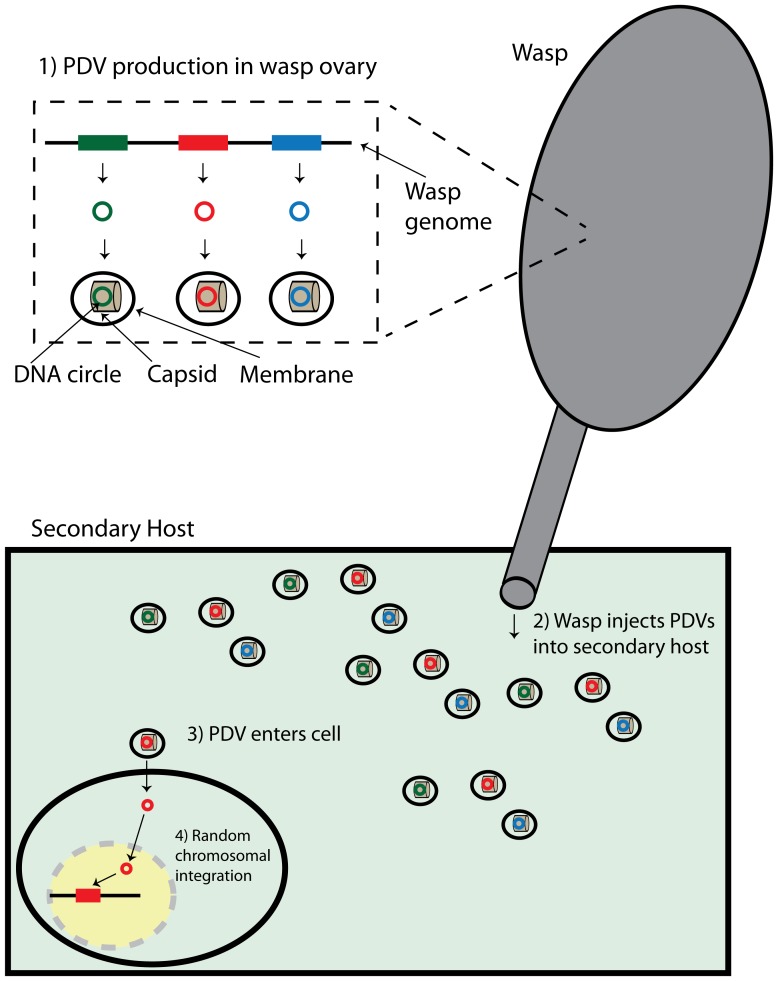
Illustration of bracovirus production and integration. In part 1 specific portions of the wasp genome are targeted, replicated, and circularized into bracovirus DNA circles. These circles are produced in the wasp ovary then packaged in a viral capsid and lipid membrane. In Part 2 the bracoviruses are injected into the secondary host. In part 3 the bracovirus enters the secondary host cell, shedding the capsid. This typically occurs in haemocytes, with the proteins produced by the transferred DNA destroying or disabling the haemocyte's ability to defend the secondary host against wasp larvae. Occasionally, bracovirus DNA will integrate into the secondary host nuclear DNA as shown in part 4. If steps 3 and 4 occur in a germ line cell the bracovirus DNA may be passed on to the secondary host's offspring. Subsequent genetic drift or selection can result in the HTS becoming fixed in the population.

Specific portions of the wasp's genome are packaged [12] into the virus-like particles (see Figure 1), but genes encoding viral functions are not included, leaving PDV particles without the molecular machinery to replicate in the secondary host. An important evolutionary consequence of the mechanism of PDV production is that the propagation of PDVs depends exclusively on survival of the wasp eggs growing in the secondary host. As a result, suppression of secondary host immunity is crucial to the survival of the wasp/virus mutualism. PDV genomes encode a number of mechanisms to suppress secondary host immunity, such as controlling gene expression with modified histones [17], interfering with the haemocyte cytoskeleton [18], and inhibiting phagocytosis [19]. In addition to suppressing the host immune system, PDV genes can also function to shift resources from secondary host larval development to growing wasp larvae [20]. A number of cystatins in *Cotesia* bracoviruses are thought to suppress the development and immune response of the secondary host [21]. These cystatin genes have been under recent positive selection, consistent with classical host/parasite evolution [22].

Despite the many mechanisms PDVs employ to attack the host, these parasitoid attacks vary widely in lethality depending on their timing. Ultimately, a secondary host must survive a PDV attack and reproduce for a horizontally transferred sequence to be seen in the population. In one study, infection by a bracovirus quickly after the start of the fifth instar (the final stage of development before metamorphosis in the species) resulted in a 100% lethality rate, while infection 60 hours post-ecdysis (the shedding of the old cuticle) had 0% lethality and generally had little effect on the larvae [23]. These experiments illustrate the possibility of secondary host survival with a foreign sequence after PDV infection.

PDVs have now been sequenced from several wasps, [24], [25] including many related braconid wasps in the *Cotesia* family [26], [27], [28]. Analysis of *Cotesia* relatives reveals that many of the genes packaged into PDVs are part of large gene families. These genes are eukaryotic in origin and some of these gene families only appear to be found in wasps [28]. In addition to positive selection acting on coding sequences, these gene families also appear to be subject to birth-death evolution typical of large gene families under strong or quickly changing selection [29].

Bracoviruses and ichnoviruses arose independently from different viruses early in the evolution of Braconid and Ichneumonid wasps [30]. Braconid wasps originated around 70–100 MYA [31], [32], [33] when a nudivirus stably integrated into the germ line of an ancestral braconid wasp [34], [35]. The origin of ichnoviruses is less clear. Reflecting their different origins, the two groups have different viral gene content and structure. Ichnoviruses package a suite of dsDNA circles in each virion particle, while bracoviruses only include one (of many) circles per virion particle [36]. This means different bracovirus particles from the same wasp have different contents (see Figure 1) and that it is possible for a wasp to inject more of one dsDNA circle than another [37].

The fact that PDVs routinely transfer wasp DNA to the secondary host has prompted researchers to investigate how long this DNA persists and whether it can permanently integrate into the secondary host [38], [39], [40]. Bracovirus DNA circles from the wasp *Cotesia congregata* were shown to persist for at least 6 days throughout the living body of the secondary host, *Manduca sexta* [41]. Additionally, Lepidopteran cell cultures infected with PDVs show stable integration into chromosomes and expression of both bracoviruses [42] and ichnoviruses [43]. Interestingly, both studies found biased integration favoring some PDV segments over others. What has been absent until now is a demonstration that this type of integration can occur outside of laboratory cell cultures, in living organisms.

## Results

The initial screen of this study was an extensive bioinformatic search for polydnavirus sequences in eukaryotic genomes. In our initial screen there were 18,797 hits in total before any filters were applied. Within these, 99.44% of our tblastx matches were found in invertebrates even though the invertebrate genomes were only 65/165 (39.4%) of the genome assemblies queried (and an even smaller fraction of the sequence space searched). In the remaining 0.56% of tblastx matches there were 101 vertebrate matches and 11 plant matches. The invertebrate matches had an average tblastx score of 116.4 (e-values 1E-06 to 1E-200) while the non-invertebrates had an average match score of 58.3(e-values 3E-07 to 5E-024). As a result of being poor matches, 88.4% of the non-invertebrate matches were removed by the initial length (67 amino acids) and tblastx score (minimum 70) thresholds alone, with the rest being removed by later filters. In contrast, 31.3% of invertebrate hits were filtered out by those thresholds.

After applying a series of quality filters (see methods), our bioinformatic search revealed many candidate PDV-derived horizontally transferred sequences (HTS) in two Lepidopteran species (Table 1). This is consistent with the natural host range of these wasps, which is heavily enriched for targeting Lepidoptera larvae. These sequence similarities violate the known insect phylogeny and suggest horizontal gene transfer.

**Table 1:** Summary information for candidate horizontally transferred sequences.

PDV#	homology group	host species	donor PDV	sequence length	%id	e-value	dn/ds	Pfam	PCR tested
1	1	bmor	NC_006648.1	349	88.78	2E-76	0.5845	-	-
2	1	bmor	NC_006637.1	349	86.67	3E-72	0.8643	-	-
3	1	bmor	NC_006637.1	351	86.2	5E-71	1.0603	-	-
4	1	bmor	NC_006648.1	376	87.84	2E-70	4.2836	-	-
5	1	bmor	NC_006648.1	343	87.84	2E-70	2.0831	-	-
6	1	bmor	NC_006648.1	341	86.89	2E-70	3.2836	-	-
7	1	bmor	NC_006648.1	336	86.89	2E-70	2.7673	-	-
8	1	bmor	NC_006637.1	341	86.97	8E-70	2.7586	-	-
9	1	bmor	NC_006648.1	340	86.85	8E-70	3.306	-	-
10	1	bmor	NC_006648.1	339	86.81	3E-69	3.3829	-	-
11	1	bmor	NC_006648.1	339	86.81	3E-69	3.3829	-	-
12	1	bmor	NC_006648.1	339	86.81	3E-69	3.3828	-	-
13	1	bmor	NC_006648.1	339	86.81	3E-69	3.3829	-	-
14	1	bmor	NC_006648.1	339	86.81	3E-69	3.3829	-	-
15	1	bmor	NC_006648.1	339	86.81	3E-69	3.3829	-	-
16	1	bmor	NC_006648.1	311	87.58	3E-69	2.4064	-	-
17	1	bmor	NC_006648.1	339	86.81	3E-69	3.3829	-	-
18	1	bmor	NC_006648.1	339	86.81	3E-69	3.3829	-	-
19	1	bmor	NC_006648.1	341	86.81	3E-69	3.508	-	-
20	1	bmor	NC_006648.1	299	87.54	1E-68	2.4938	-	-
21	1	bmor	NC_006648.1	346	87.5	5E-68	1.3091	-	-
22	1	bmor	NC_006648.1	339	87.5	5E-68	2.3231	-	-
23	1	bmor	NC_006648.1	340	86.54	2E-67	3.1067	-	-
24	1	bmor	NC_006648.1	347	86.54	2E-67	3.4075	-	-
25	1	bmor	NC_006648.1	339	86.5	8E-67	3.5527	-	-
26	1	bmor	NC_006648.1	339	86.5	8E-67	2.6987	-	-
27	1	bmor	NC_006648.1	341	86.5	8E-67	3.4834	-	-
28	1	bmor	NC_006648.1	339	86.5	8E-67	3.6816	-	-
29	1	bmor	NC_006648.1	341	86.5	8E-67	3.4834	-	-
30	1	bmor	NC_006648.1	339	86.5	8E-67	3.3829	-	-
31	1	bmor	NC_006648.1	321	87.16	1E-65	3.1914	-	-
32	1	bmor	NC_006648.1	559	87.33	2E-65	1.2302	-	yes
33	1	bmor	NC_006648.1	299	87.16	1E-65	2.2366	-	-
34	1	bmor	NC_006648.1	340	86.24	5E-65	2.594	-	-
35	1	bmor	NC_006648.1	352	87.25	5E-65	0.9559	-	-
36	1	bmor	NC_006637.1	357	86.36	5E-65	1.9572	-	-
37	1	bmor	NC_006648.1	370	86.15	8E-64	1.794	-	-
38	1	bmor	NC_006648.1	282	87.59	4E-62	0.7707	-	-
39	1	bmor	NC_006648.1	344	87.04	5E-62	0.8098	-	-
40	1	bmor	NC_006654.1	243	89.04	5E-61	0.0156	-	-
41	1	bmor	NC_006648.1	339	85.58	1E-59	1.2655	-	-
42	1	bmor	NC_006648.1	254	88.66	3E-59	0.7043	-	-
43	1	bmor	NC_006654.1	241	88.5	2E-57	0.0672	-	-
44	1	bmor	NC_006654.1	207	88.89	1E-55	0.3773	-	-
45	1	bmor	NC_006640.1	290	95.65	2E-54	0.971	-	-
46	1	bmor	NC_006648.1	309	85.34	9E-54	3.0969	-	-
47	1	bmor	NC_006637.1	271	85	7E-39	0.7679	-	-
48	1	bmor	NC_006648.1	243	91.51	3E-31	2.7586	-	-
49	1	bmor	NC_006637.1	236	96.83	2E-23	0.6128	-	-
50	2	danple	HQ009532.1	390	88.8	2E-67	0.1749	-	-
51	2	danple	HQ009532.1	385	88.52	9E-67	1.0483	-	-
52	2	danple	HQ009532.1	376	88.61	5E-65	1.3172	-	-
53	2	danple	HQ009530.1	851	85.8	1E-61	0.4301	-	-
54	2	danple	HQ009530.1	1159	85.8	2E-61	0.4964	-	-
55	2	danple	HQ009532.1	679	88.61	6E-60	0.8272	-	-
56	2	danple	HQ009530.1	436	82.92	1E-59	0.4798	-	-
57	2	danple	HQ009530.1	414	85.63	2E-58	0.3327	-	-
58	2	danple	HQ009530.1	429	84.37	2E-58	0.4604	-	-
59	2	danple	HQ009532.1	382	88.57	5E-56	1.8185	-	-
60	2	danple	HQ009532.1	270	87.22	1E-55	0.2903	-	-
61	2	danple	HQ009532.1	697	88.68	1E-54	81.7186	-	-
62	2	danple	HQ009532.1	267	88.13	2E-54	0.3171	-	-
63	2	danple	HQ009530.1	275	85.29	8E-54	0.7488	-	-
64	2	danple	HQ009530.1	285	84.89	1E-52	0.3398	-	-
65	2	danple	HQ009532.1	382	89.06	2E-52	0.7265	-	-
66	2	danple	HQ009530.1	207	88.44	1E-51	1.0305	-	-
67	2	danple	HQ009532.1	241	87.11	7E-51	0.2693	-	-
68	2	danple	HQ009532.1	313	88.29	9E-51	0.0696	-	-
69	2	danple	HQ009530.1	278	85.66	1E-49	0.678	-	-
70	2	danple	HQ009530.1	209	87.8	1E-48	1.1323	-	-
71	2	danple	HQ009530.1	224	88.12	2E-48	0.6006	-	-
72	2	danple	HQ009530.1	297	86.35	2E-48	1.4229	-	-
73	2	danple	HQ009530.1	252	86.55	3E-47	0.4727	-	-
74	2	danple	HQ009532.1	282	87.69	3E-47	0.4589	-	-
75	2	danple	HQ009530.1	262	84.73	1E-46	0.2809	-	-
76	2	danple	HQ009530.1	265	88.36	1E-46	0.4926	-	-
77	2	danple	HQ009530.1	291	87.44	5E-46	0.7242	-	-
78	2	danple	HQ009530.1	262	86.55	1E-43	0.9544	-	-
79	2	danple	HQ009530.1	261	93.16	3E-41	0.2191	-	-
80	2	danple	HQ009530.1	233	89.81	2E-41	0.4806	-	-
81	2	danple	HQ009530.1	252	93.16	3E-41	0.3233	-	-
82	2	danple	HQ009530.1	254	87.43	1E-40	1.5364	-	-
83	2	danple	HQ009530.1	227	88.55	4E-40	0.431	-	-
84	2	danple	HQ009530.1	268	84.29	2E-39	0.9501	-	-
85	2	danple	HQ009532.1	226	85.78	5E-39	1.2444	-	-
86	2	danple	HQ009530.1	290	84.38	3E-38	0.4706	-	-
87	2	danple	HQ009530.1	241	89.51	9E-38	0.3117	-	-
88	2	danple	HQ009530.1	208	85.65	8E-38	0.4756	-	-
89	2	danple	HQ009530.1	263	90.68	1E-34	0.4903	-	-
90	2	danple	HQ009530.1	264	83.26	9E-26	0.7161	-	-
91	3	bmor	HQ009550.1	237	85.71	3E-47	0.494	-	-
92	3	bmor	HQ009550.1	224	85.12	6E-42	0.961	-	-
93	3	bmor	HQ009550.1	212	82.3	4E-24	0.9383	-	-
94	4	danple	NC_006654.1	6570	88.46	1E-200	0.7277	BEN, Pfam-B_3333	-
95	4	danple	NC_006654.1	4091	89.55	1E-200	1.005	-	-
96	4	danple	NC_006660.1	2188	90.33	1E-200	0.857	BEN	-
97	5	danple	NC_006660.1	409	91.15	7E-154	0.303	-	-
98	5	danple	NC_006660.1	339	91.67	3E-131	0.3989	-	-
99	6	bmor	HQ009550.1	207	85.94	4E-24	1.1119	-	yes
100	7	bmor	NC_006645.1	415	87.9	2E-64	0.6016	-	yes
101	8	bmor	NC_006644.1	1918	87.68	1E-200	1.054	-	yes
102	9	danple	NC_006633.1	891	92.39	6E-110	0.7112	-	-
103	10	danple	HQ009530.1	245	82.38	3E-22	0.6184	-	-
104	11	danple	HQ009545.1	338	82.52	2E-48	0.4731	-	-
105	12	danple	NC_006639.1	1547	87.72	1E-200	1.5257	-	-

Summary information for PDV HTS. Column labeled “PDV#” shows the identification number to the PDV HTS used in this manuscript. “homology group” gives the homology group the HTS belongs to. “host species” indicated the host genome the HTS was found in. “donor PDV” gives the PDV sequence the HTS matches to. “sequence length” is the length, in nucleotides, of the HTS. “%id” is the percentage nucleotide identity between the HTS found in the host and the donor PDV. “e-value” is the e-value from the blastn screen. “dn/ds” is the pairwise ratio of non-synonymous protein changes to synonymous protein changes. “Pfam” shows protein family matches for the sequence. “PCR tested” shows if the sequence was tested by PCR.

To test whether these matches could be explained by unknown insect genes giving a false signal resembling horizontal gene transfer, several analyses were performed. The candidate HTS were used as queries in a search against a combined database with the same 165 eukaryotic genomes, the 387 PDV sequences, and NCBI's non-redundant viral sequence list(see methods). In all cases the candidates matched more closely to PDVs. The same search was performed by web-BLAST against NCBI's non-redundant database and all the candidates matched best to PDVs.

In an additional analysis, each of the remaining HTS candidates were searched against the set of 165 eukaryotic genomes with the goal of finding other sequences related to the HTS. In every case, no new matches were found. This indicates that the only sequences with significant sequence similarity to the HTS candidates in the two Lepidopterans are wasp PDV sequences. Thus, our reported 105 HTS are each only found in two places: the Lepidopteran genome assembly (either *Bombyx mori* or *Danaus plexippus*) and the PDV assembly from a braconid wasp.

**Figure 2:**
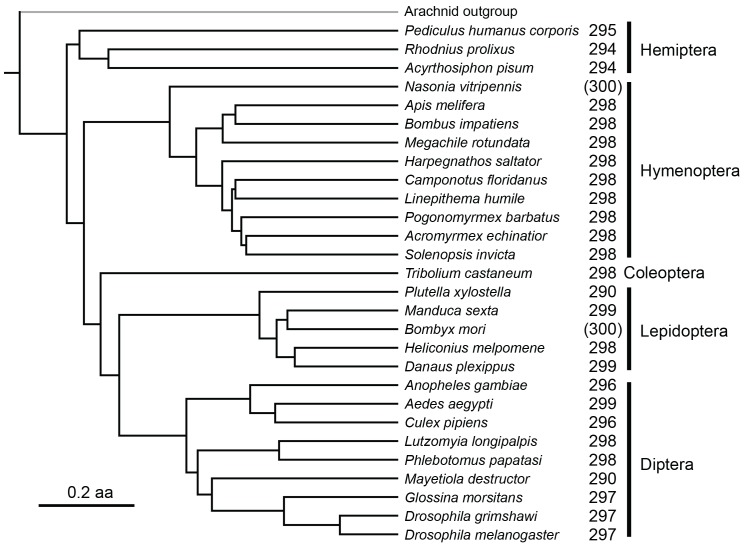
Representation of highly conserved sequences in insect genomes. Phylogenetic tree generated from sequence data using PHYML. Coding regions from *Nasonia vittripennis* were used as tblastx queries against the *Bombyx mori* genome assembly to identify highly conserved regions. 300 conserved regions were selected at random and searched for in a broad range of insect genome assemblies using tblastx. In each insect species, 290–299 of these highly conserved regions were found (a rate of 96–99%). Missing sequences are an unknown combination of genomic deletions and incomplete assemblies. With a divergence time of ∼300 MYA between *Bombyx* and *Nasonia*, there has been sufficient time for genomic deletions to occur. Branch lengths are based on amino acid divergence between species.

This distant distribution is quite unusual. Sequences with a high relatedness between Lepidoptera and Hymenoptera typically come from highly conserved genes with a broad distribution among insects. In an additional analysis, we identified 2,631genes from the wasp *Nasonia vitripennis* with homology to genes in *Bombyx mori*. 300 of these were selected at random to perform a phylogenetic analysis examining how frequently these genes could be found in other insect species. We found that these random *Nasonia* genes are present in 96–99% of insect assemblies (Figure 2). This is dramatically different from the sequences examined in this manuscript, which are only found in a single species (plus the donor wasp). We also measured the degree of nucleotide conservation for each gene between the wasp copy and the copy in the other insect. The tblastx match regions between *Bombyx* and *Nasonia* for these 2,631 genes had median length 537 nucleotides (range 183–2745) and median 71.3% nucleotide identity (range 58.5–87.3). We found a lower degree of conservation amongst these randomly selected wasp genes (median 71.3%) than in our HTS candidates (median 86.95%). The 105 HTS candidates were found to have significantly different percent identities than the 2,631 conserved *Nasonia* genes found in *Bombyx* using the Mann-Whitney u-test (p<2.3E-09). All 300 genes had a broader phylogenetic distribution than any of the PDV matches. The combination of the candidate HTS only being found in one very distant species and having a higher nucleotide identity than conserved genes is indicative of these candidate sequences being horizontally transferred. None of the HTS found in *Bombyx mori* had any homology to HTS in *Danaus plexippus* and vice versa, indicating separate integration events.

**Figure 3:**
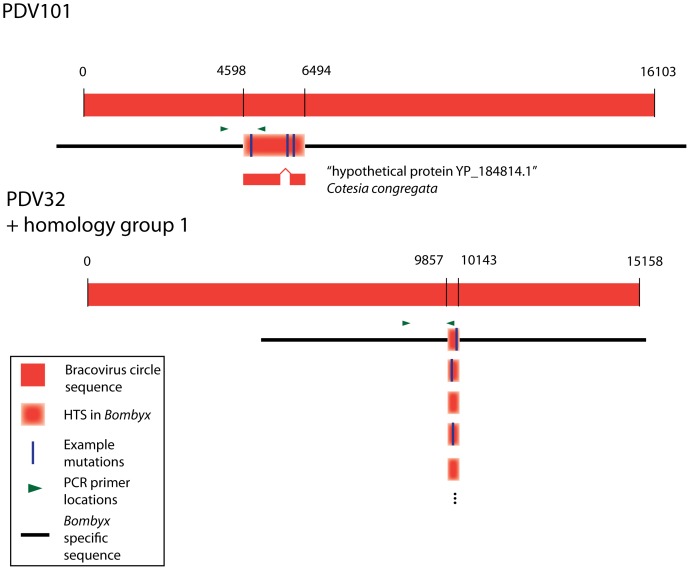
Two examples of HTS found in *Bombyx* (the same two HTS targeted for PCR in **Figure 4**) aligned to bracovirus sequences from *Cotesia congregata*. The top portion shows a HTS (PDV101) aligned to *Cotesia congregata* bracovirus circle 12 (NC_006644.1). Predicted protein in the donor sequence shown (YP_184814.1). The bottom portion show a HTS (PDV32) aligned to *Cotesia congregata* bracovirus circle 5 (NC_006637.1). PDV32 is part of homology group 1, which is shown aligned to PDV32. In both examples the HTS have undergone point mutations and insertion/deletions that are too numerous to accurately represent here so example mutations have been drawn. Primers used in the PCR reaction shown in Figure 4 are shown with green triangles.

The HTS were sorted into homology groups, with a member being added to a group if it had 90% similarity over 90% of its length to another member of the group (HTS are listed with groups in Table 1). Some of these HTS exist as a single copy while others appear to be part of such groups (Figure 3). Homology groups containing more than one sequence were aligned by DIALIGN [44] and placed onto trees using PHYML [45] (Figure S1). Those that appear together in a homology group are the result of an unknown mix of two factors: repeated integration of the same PDV locus (circle) into the secondary host and subsequent duplication of the sequences in the secondary host. Many repeated integrations of the same sequence are expected from previous cell culture studies [42], [43]. It is possible that there are mis-assemblies in the genome sequences that affect the apparent copy number of these sequences. This could increase or decrease the copy number by splitting alleles into paralogs or vice versa. For these reasons it is difficult to know exactly how many copies exist in the destination genomes, though it must be at least one copy to show up in the assembly. Because the destination genome assemblies do not have chromosome information, only small contigs, there is no information regarding whether the transferred sequences preferentially integrate into certain portions of the destination genome.

Three PDV queries (NC_006658.1, HQ009558.1, EF067323.1) were found to contain significant matches to transposable elements (a gypsy, helitron, and Jockey respectively). Hits matching these sequences were discarded for clarity and focus in this manuscript. However, it is worth noting that if the central hypothesis of this paper is correct (that PDVs will tend to transfer genomic sequences from parasitoid wasps to their host species) it would predict that transposable elements like these would frequently make the transfer from wasp to host along with their surrounding genomic DNA. If a transposable element is targeted for packaging into a PDV, it will be among a small portion of the genome that is heavily enriched, copied in large numbers, and transferred to the host species making PDVs a strong vector for spreading of transposable elements. Once transferred to the host species, these could expand in number and spread throughout the new genome.

To gain insight into the function of ORFs in candidate HTS, we searched the translated protein sequence of the HTS against the PfamA and PfamB databases (http://pfam.sanger.ac.uk/search) using a highly permissive e-value (1e-03). Two sequences (PDV94 and PDV96) were found to contain the BEN domain, which is known to be found in PDVs and is associated with growth arrest and transcriptional regulation. PDV94 also matches to the protein family Pfam-B_3333 (function unknown). The function of ORFs in other candidate HTS is unknown.

Based on previous work, we expect that PDV loci transferred to secondary hosts were under recurrent positive selection before being transferred [25], [46]. To test for positive selection in coding regions, the HTS were aligned by codon to the matching PDV sequence and then analyzed for synonymous and non-synonymous changes by maximum likelihood using PAML [47]. The results indicate that many of the sequences have a dn/ds>1, suggesting that they have undergone recent positive selection (Table 1). A dn/ds <1 generally indicates that a sequence is conserved, though it does not rule out the possibility of positive selection on a small number of residues in the protein. It is possible that the positive selection we observed occurred prior to transfer [25], [46], however it is also possible that some of these sequences were co-opted and selected upon after transfer to the secondary host as well.

**Figure 4:**
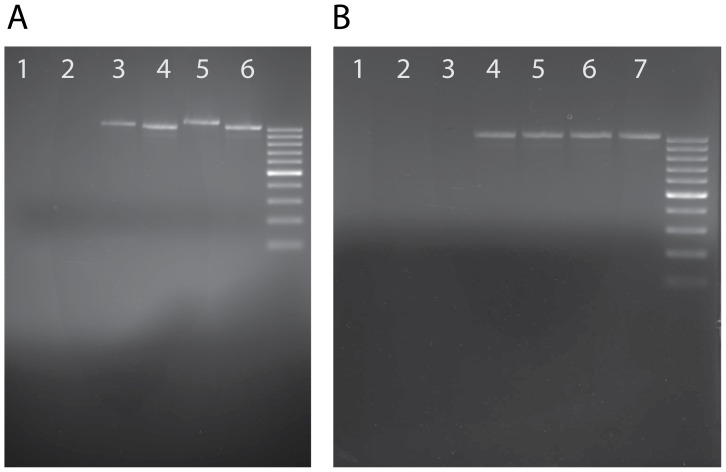
Gels displaying PCR amplification of HTS in *Bombyx mori*. Gels were ethidium bromide stained and run with a 100 bp ladder (brighter bands at 500 bp and 1000 bp). Primers used are shown in fig 3. As a further test, additional PCR reactions were run with alternative primers for each HTS with the same results (see Figure S2). **A**) PCR results showing amplification of a product spanning the junction of the transferred wasp DNA and native *Bombyx* sequence. Lanes 2, 4, and 6 have primers targeting PDV32 (expected band size: 1037) and lanes 1, 3, and 5 have primers targeting PDV101(expected band size 1090). Lanes 5 and 6 use *Bombyx mori* strain 418(Chinese), lanes 3 and 4 *Bombyx mori* strain 214(Japanese), lanes 1 and 2 wild type *Drosophila melanogaster* as a negative control. **B**) We tested the same primer set above targeting PDV32 against a diverse panel of insect genomic DNA (all lanes tested with the same primers). DNA used in the reactions was as follows. Lane 1: *Chlosyne lacinia* (Lepidoptera). Lane 2: *Apis mellifera* (honeybee, Hymenoptera). Lane 3: wild type *Drosophila melanogaster* (Diptera). Lane 4: *Bombyx mori* strain 555 (European). Lane 5: *Bombyx mori* strain Nistari (Indian). Lane 6: *Bombyx mori* strain 418 (Chinese). Lane 7: *Bombyx mori* strain 214 (Japanese).

To rule out the possibility of an apparent HTS being the result of genome assembly error, the presence of HTS were tested by PCR directly from Bombyx genomic DNA with DNA from other insects used as a control (Figure 4). Two representative HTS (PDV32 and PDV101) from *Bombyx mori* were arbitrarily selected: one larger sequence that appears a single time and one smaller sequence that appears many times in the assembly. Both HTS successfully amplified from all *Bombyx* strains tested and did not amplify in control species, confirming that these sequences are not artifacts of the *Bombyx* sequencing project. Additional PCR amplifications were performed on the same two HTS with alternate primers and similar results obtained and PCR amplifications of HTS100 and HTS99 were performed, amplifying in all strains tested (see Figure S2).

## Discussion

While previous work has shown a strong trend for HGT between parasitic species and host species [4], [5], [6], [7], [8], the findings presented here are the first known instances of HGT between a *parasitoid* species and host species. Most parasites typically do not kill the host they feed upon, making them a passive vector for horizontal transfer if there happens to be a relevant infection by a vector species (e.g. retrovirus). Unlike parasites, successful parasitoids ultimately kill their host, meaning the secondary host must also defeat the parasitoid infection and survive to reproduce for HGT to occur. Parasitoid/PDV attacks vary widely in lethality. Attacks earlier in development can have a 100% lethality rate while attacks as little as 60 hours later can have 0% lethality and few effects [23]. Based on this, it seems likely that the ancestral infections discussed in this manuscript occurred in larvae that were at a later stage of development. This window of time in which PDV infections can occur but have little effect on the host provides a natural avenue for HGT.

Those parasitoids that utilize polydnaviruses likely increase the chance of horizontal transfer by many orders of magnitude by enriching a small portion of their genome and injecting it directly into the host in a specialized viral vector. Combined with the variable lethality of PDV infections, this gives a plausible range of natural circumstances in which horizontal transfer can occur. Previous work has shown this potential for PDVs to facilitate HGT in somatic insect cell lines, but never in germ line cells or living organisms in natural populations. Our findings represent the first documented cases of HGT using a polydnavirus vector in a live organism.

Though the mechanisms by which PDVs integrate into live organisms are poorly understood, this HGT raises the possibility of experimentally manipulating the system. These PDVs could be used as gene delivery vectors for the many plant [48], [49] and animal species these viruses naturally affect, and possibly others. The targeting and efficiency of gene transfer using PDVs could be improved, making PDVs a potentially useful vector that will naturally self-terminate due to a lack of reproductive capability. If PDVs could be produced from wasp ovary cell cultures, this targeted gene transfer could be achieved using artificial injections without rearing wasps or infecting hosts with wasp larvae.

One interesting feature of these PDV producing wasps with respect to HGT is that, unlike many other instances of HGT from one multicellular eukaryote to another, there is no independently reproducing third species required to act as an intermediary. In many other instances of eukaryote HGT, this role is played by an organism (e.g. retrovirus) which is free living and then randomly acquires and carries the sequence from the donor species for a time, often replicating it as a part of its genome, until infecting the recipient species and transferring the sequence to the recipient germ line. HGT facilitated by PDVs is quite different. In this case everything needed to complete the HGT is contained within the wasp/host system. On the donor side, the sequences targeted for packaging in PDVs is non-random: a small subset of the wasp genome is replicated many times and packed into the PDV vector. On the recipient side, the host is a species specifically targeted by the wasp (and not an undirected infection like in the case of other vectors).

These differences predict several effects we expect to see when HGT is facilitated by PDVs. One is that there should be an increased number of horizontal transfers going from wasp to host. Another prediction is that the transferred regions should be regions targeted for PDV packaging. Because PDVs cannot replicate independently, another prediction is that these transferred sequences would not be expected to be found in species that are not targeted by these wasps. In the case of other vectors, the same virus that infects the recipient's germ line could continue reproducing and infect many other organisms (and species), producing a different, more dispersed pattern of HGT than one expects with PDVs.

This unusual genetic phenomenon creates an evolutionary paradigm in which a host species can acquire genetic information from a parasitoid species that attacks it. Previous work has discussed the acquisition of sequences from viral pathogens [50], [51], [52], [53] however discussion of the acquisition of sequences from eukaryotic parasitoids has been absent up to this point. As with mutations or other horizontally transferred sequences, these sequences have the potential to be adaptive, maladaptive, or neutral for the host (probably more often neutral/maladaptive). Maladaptive sequences are typically removed over time by selection, so sequences found in extant organisms are likely depleted for maladaptive sequences. Occasionally, transferred sequences could be co-opted and adapted by the host in a number of ways. The chance of a sequence being co-opted would be expected to increase with the number and diversity of transferred sequences. The ways that the sequences could be adapted to the new host include controlling the host's gene regulation, altering its physiology, or using those sequences as a direct countermeasure against wasp parasitization to fight future PDV infections. For example, the secondary host could use the transferred sequences to produce anti-sense RNA to mark transcribed PDV mRNAs for degradation by normal processes that affect double stranded RNA. Another possibility is that the secondary host could co-opt protein interacting domains taken from the wasp to bind and block proteins produced by the virus. The secondary host could also find completely novel uses for the sequences, such as regulating its growth or immune system. Since it is estimated that there are hundreds of thousands of PDV producing wasp species (and their corresponding host species, ranging from insects to plants), our findings are likely a first look at a large and unusual phenomenon. This sort of horizontal transfer is likely to be discovered more frequently as more eukaryote species and PDVs are sequenced.

Tracking PDV HTS in secondary hosts can be a useful tool for evolutionary studies. It should be possible to deduce parasitoid/host relationships, potentially even if there is no known parasitoid affecting the secondary host species. This is the case with the data presented here, which predict a parasitoid which affected *Bombyx mori* (or an ancestor species) though none is currently known. Given the moderate sequence divergence observed between the HTS found in *Bombyx/Danaus* and the donor PDVs, this suggests that the transfers were from some ancestors of *Cotesia* wasps to some ancestors of *Bombyx/Danaus* and may not involve any presently extant species. Evolutionary analysis of PDV HTS is applicable not just to present parasitoids but also parasitoids from the past, opening up the exciting possibility of tracking relationships that involve extinct species.

## Methods

### BLAST searches

The initial screen of this study was an extensive tblastx search of 387 PDV sequences against 165 eukaryote genomes including 75 vertebrate species, 25 plant species, and 65 invertebrate species. Among the invertebrates, there are 48 insect species including 15 Hymenoptera, 5 Lepidoptera, and 24 Diptera. A complete list of PDV queries and eukaryotic whole genome assemblies can be found in Table S1.

PDV sequences contain an unknown mix of coding sequence, regulatory sequence, introns, intergenic regions, and repeat content. When a PDV is naively searched against whole genome sequences, any of these features could match to the whole genome sequence. As a result, matches could appear in species that are not known to be parasitized by PDV wasps (e.g. mammals). These could be caused by simple repeat sequences or by proteins common to the larger group (e.g. metazoans). Tblastx was used because it is highly sensitive at detecting evolutionarily divergent proteins with different amino acids but conserved function (e.g. substituting a non-polar amino acid for a different non-polar amino acid) using the BLOSUM62 matrix. Sequences from this screen were kept if they had all the following: an e-value below 1.0e-10, a BLAST score greater than 70, and a match length of at least 67 amino acids. Remaining hits found on the same contig nearby one another (<5 kb) were merged into a single hit.

Using these ORF containing protein matches as initial regions, we expanded our investigation to regions surrounding these open reading frames to get a more complete understanding of how much sequence was transferred. The protein match from the PDV sequence plus 5 kb of flanking DNA in both directions were searched against the destination genomes for a strong match in DNA sequence. Portions of these ORF/extension regions that match to the destination genome could indicate a large stretch of DNA transferred by PDV to the destination genome. Sequences in this search were kept if they had the following: a match length of at least 200 nucleotides and a percent identity of at least 80%. The remaining sequences were our initial pool of potentially horizontally transferred sequences.

### Removing repeats

Sequences matching a PDV sequence found to contain a transposon (NC_006658.1, HQ009558.1, EF067323.1) were removed. While we expect transposons to be transferred from PDVs to destination genomes just as a coding sequence would, they were removed for clarity and focus in this manuscript. Transposons were found by searching the 387 PDV sequences with RepeatMasker 3.3.0 (default settings). PDV sequences with significant matches to transposons were removed from the study.

Remaining candidate sequences were filtered for simple repeats. The DNA sequence of each candidate was searched by RepeatMasker 3.3.0 for significant matches with the following settings: -noint, –poly, default settings elsewhere. Any candidate with a significant match to simple repeats was removed.

### Homology Analysis

The set of candidate sequences in the destination genomes were then analyzed and placed into homology groups. All the candidate sequences were searched against themselves using blastn with a max e-value of 1.0e-10. Candidate sequences were placed in a group together when a sequence has a 90% nucleotide identity over 90% of its length to any member in the group. Homology groups (and their candidates) containing more than one destination genome were removed from the study. Though such sequences may be horizontally transferred, we removed the sequences on the possibility that they could be vertically transmitted. There is no biological mechanism precluding protein sequences shared by metazoans or eukaryotes being included in a PDV. If such a shared sequence is included in a PDV, our screen for protein matches would find them and report a dispersed pattern of hits across many destination genomes. Because of this concern, homology groups showing that pattern were removed from the study.

In addition to searching for homology groups within our candidate transferred regions (above), we also searched for homology between our candidate sequences and the whole genome sequences of a large set of organisms. Specifically, we searched the candidate HTS against a database including our set of 165 eukaryotic genomes, the original 387 PDV sequences, and NCBI's non-redundant virus list (containing “conventional” viruses). In each search the strongest match of all the sequence space searched (whether viral, PDV, or eukaryote) was recorded. A sequence that matches more strongly to any eukaryotic sequence than to a PDV or virus sequence (self-hits excluded) could indicate that the sequence is an insect gene shared between those species by a distant common ancestor and not actually a PDV horizontally transferred sequence. If the sequences in the destination genomes are truly horizontally transferred regions, they should match more strongly (>20 blast score gap) to PDV sequences than to any other sequence. Searches were also performed by web-BLAST against NCBI's non-redundant database.

### Testing conserved insect regions

We started with the coding regions of known Bombyx mori mRNAs (ftp://ftp.ncbi.nlm.nih.gov/genomes/Bombyx_mori/RNA/) and used each as a tblastx query to the parasitic wasp *Nasonia vittripennis* genome assembly. *Bombyx* coding genes that had a *Nasonia* match score with the threshold used in the PDV searches (BLAST score 70 or higher) were extracted and a single splice form was retained for each such gene. This produced a set of 2,631 *Bombyx* genes that include a highly conserved segment present in the *Nasonia* genome. We selected 300 random genes from these 2,631 and used the same tblastx test to determine the representation of such highly conserved gene segments across a representative set of 26 insect genome assemblies. Results of the representation of these genes among tested insect genomes are shown in Figure 2, mapped onto a phylogenetic tree of the species tested.

The phylogenetic tree in Figure 2 was constructed as follows. A suite of 1,400 *Apis mellifera* coding exons that are present in single copy widely in insects was obtained, starting with all the coding exons annotated by Ensembl Amel4.0, and using tblastn to eliminate exons that are absent or present in more than one copy in the *Apis mellifera* (Amel 4.5 assembly), *Tribolium casteneum* (Tcas3.0 assembly), *Drosophila melanogaster* (dm3 assembly), or *Ladonia fulva* (Lful_1.0 assembly) genomes. This suite of exons was used as query in tblastn searches of all the species shown in Figure 2 plus the Arachnid *Ixodes scapularis* (IxscaW3 assembly) for rooting. The single best tblastn match for each genome was extracted and those that were reciprocal best blast matches to the correct *Apis mellifera* coding exon were retained for alignment and tree building. For each exon, muscle3.8.31 (default parameters) [54] was used to generate a multiple protein alignment, and positions in the alignment with more than 10% gaps were removed. All the exon alignments were then concatenated to generate a large protein multiple alignment (average of 59,188 amino acids per species). This alignment was used to construct a maximum-likelihood phylogenetic tree with phyml (LG model, 6 rate classes, SPR moves). The tree obtained agrees well with all recent analyses of these species [55], [56], [57], though no single published tree contains this exact set of species.

### PCR

Two HTS were tested by PCR amplification. Primers were designed such that the reverse primer was placed in a region alignable between the wasp PDV sequence (see Fig. 3) and the Lepidopteran sequence (i.e. having strong homology between the sequences). The forward primer was placed in a region that was unalignable between the wasp and Lepidopteran sequences (regions lacking homology). Thus the PCR product spanned the junction between the transferred regions and non-transferred regions. PCR reactions where both primers were placed on transferred sequence were also performed, and are shown in Figure S2.

The PCR product was run on a 1.5% agarose gel with a 100 bp ladder and stained with ethidium bromide. The 20 ul PCR reaction contained: 5 ul genomic DNA at 30 ng/ul, 5 ul New England Biolabs 5× taq master mix, 1 ul fwd primer, 1 ul rev primer, 8 ul H20.

Primers are drawn on the relevant sequences in Figure 3 and detailed below. Gels shown in Figure 4.

#### PDV32

Forward primer 5′-TTTCACCATCGTCTCGTCCC-3′ (nscaf2876: 1094875–1094894). Reverse primer 5′- AGGCAGCTGGTTGTGAACAG-3′ (nscaf2876: 1095911–1095892). Expected product size: 1037.

#### PDV101

Forward primer 5′- AGCAACGTGAGAACTCTACGAA-3′ (nscaf3026: 4678131–4678110). Reverse primer 5′- AGGCAGCTGGTTGTGAACAG-3′ (nscaf3026: 4677042–4677061). Expected product size: 1090.

Six of different strains of *Bombyx mori* were tested for the presence of these hits (all strains courtesy of Marian Goldsmith, University of Rhode Island). **p50**: Inbred Chinese-originating strain used in sequencing by Japanese part of *B. mori* sequencing project. Originated from lab of Toru Shimada, University of Tokyo. **Nistari**: a multivoltine (multiple generations per year; no diapause) strain originally from India then brought to an INRA lab in Lyon. **401**: Chinese strain reported to have BT-resistance. **418**: Chinese strain. **214**: Japanese strain. **555**: European strain.

Genomic DNA from other insect species was also used as a negative control. Wild type *Drosophila melanogaster* was obtained from Celeste Berg. *Apis melifera* and *Chlosyne lacinia* were obtained from Charles Laird.

## Supporting Information

Figure S1 **DNA trees for homology groups containing more than one member.** Each tree includes all members of the homology group and the original PDV sequences that the members of the homology group matched to. Alignments were performed using DIALIGN in “genomic DNA” mode and trees were created using PHYML A) Homology group 1 B) Homology group2 C) Homology group 3 D) Homology group 4 E) Homology group 5.
(PNG)

Figure S2 **Gels displaying PCR amplification of HTS in** ***Bombyx mori*** **.** Figures S2A and S2B show alternative PCR primers amplifying the same regions as the primers used in Fig 4. Figures S2C and S2D show result for primers targeting PDV100. Figures S2E and S2F show result for primers targeting PDV99 Note that in parts C and D the Bombyx strain 106 appears to have a deletion polymorphism yielding a smaller fragment than other strains. Gels were ethidium bromide stained and run with a 100 bp ladder (brighter bands at 500 bp and 1000 bp). **A**) PCR results for reaction targeting PDV 101 with alternative primers. Lanes alternate between the two different forward primers for the reaction (expected product sizes of 1049 and 686). Tested four strains with each pair of primer sets: 418 (Chinese), 214(Japanese), Nistari (Indian multivoltene), 555(European). **B**) PCR results for alternative primers targeting PDV32 (expected product size of 947). Five strains were tested: 418 (Chinese), 214(Japanese), 401(Chinese BT-resistant), Nistari (Indian multivoltene), 555(European). **C**) PCR results for primers targeting PDV100. Lane1: B. mori 214(Japanese). Lane2: B. mori 401(Chinese). Lane 3: B. mori 108(Chinese). Lane 4: B. mori 106(Chinese). Lane 5: Drosophila Melanogaster negative control. Lane 6: Apis Melifera negative control. Lane 7: Chlosynne lacinia (butterfly) negative control **D**) PCR results for primers targeting PDV100. Lane 1: Chlosynne lacinia (butterfly) negative control. Lane 2: Apis Melifera (honeybee) negative control. Lane 3: Drosophila Melanogaster negative control. Lane 4: B. mori 106(Chinese). Lane 5: B. mori 108(Chinese). Lane 6: B. mori 401(Chinese). Lane7: B. mori 214(Japanese). **E**) PCR results for primers targeting PDV99. Lane 1: B. mori 214(Japanese). Lane 2: B. mori 401(Chinese). Lane 3: B. mori 108(Chinese). Lane 4: Apis Melifera (honeybee) negative control. Lane 5: Drosophila Melanogaster negative control. Lane 6: Tenebrio molitor(mealworm) negative control. **F**) Lane 1: Tenebrio molitor(mealworm) negative control. Lane 2: Drosophila Melanogaster negative control. Lane 3: Apis Melifera (honeybee) negative control. Lane 4: B. mori 108(Chinese). Lane 5: B. mori 401(Chinese). Lane 6: B. mori 214(Japanese). **PDV101** was run with two different forward primers against a single reverse primer in part A: Reverse primer 5′-TGCGCTTTGTCTCGGATCTT-3′ (nscaf3026: 4676695–4676676). Forward primer 1 5′- AGTGATGCGGAACCAGTGAG-3′ (nscaf3026: 4676010–4676029). Expected product size  = 686. Forward primer 2 5′-TGCTGCAATTGACTAAACCGC-3′ (nscaf3026: 4675647–4675667.) Expected product size: 1049. **PDV32** was tested with a single forward and reverse primer in part B: Reverse primer 5′-CAAAACGTGCCAGAGCCAAA-3′ (nscaf2876: 1095967–1095948). Forward primer 5′-TGGCAGACGAGCTCACAAAT-3′ (nscaf2876: 1095021–1095040). Expected product size: 947. **PDV100** was tested with the following primers in part C: Forward primer 5′-TAGACACATCAGCGCAACCA-3′ (nscaf2953: 1176106–1176125). Reverse primer 5′-AGCCAGAGTACCCGTTTTCG-3′ (nscaf2953: 1176947–1176928). Expected product size: 842. **PDV100** was tested with the following primers in part D: Forward primer 5′-ACCAGACGAGCTTGTTGTGA-3′ (nscaf2953: 1175996–1176015). Reverse primer 5′-TACAGTTCCGGGAGTACGGA-3′ (nscaf2953: 1176885–1176866). Expected product size: 890. **PDV99** was tested with the following primers in part E: Forward primer 5′-TTCGACTCACGAGGAGCCTA-3′ (nscaf2734: 12767–12786). Reverse primer 5′-AGTGGGACGAAAGTTGCCAG-3′ (nscaf2734: 13515–13496). Expected product size: 749. **PDV99** was tested with the following primers in part F: Forward primer 5′-GGGCGTTAACAATGCCAAGG-3′ (nscaf2734: 12589–12608). Reverse primer 5′-CGAAAGTTGCCAGTTTCCGC-3′ (nscaf2734: 13508–13489). Expected product size: 920.
(PNG)

Table S1 **Summary information of PDV queries used and Eukaryotic whole genome assemblies used in this study.** For the left portion: “Polydnavirus queries” lists all the PDV sequences used in the search by accession number. “Wasp species” gives the species of wasp the PDV was sequenced from. “Length” is the base pair length of the PDV sequence. “Total” gives the total amount of base pairs sequenced for that species of wasp. For the right portion: “Abbreviation” gives an abbreviation for the eukaryote database used. “Eukaryote Genome” lists the eukaryote genome databases used in full.
(XLS)

Table S2 **Detailed information for PDV HTS.** The first column “PDV#” is an arbitrary numbering scheme for the HTS. “Group” sorts the found PDV sequences into related groups. “Donor wasp species” gives the wasp species that the matching PDV derives from. “matching viral sequence” is the viral sequence a match was found for. “Recipient species” is the species the transferred sequence was found in. “Contig” shows the contig of a sequence. “%id” shows the percentage of matching nucleotides reported by BLAST. “sequence length” is the length of the horizontally transferred sequence. “query start” and “query stop” are the start and stop of the match on the query sequence (virus). “database start” and “database stop” are the start and stop of the match on the database (eukaryote genome). “evalue” is the probability of the match by chance. “score” is the BLAST alignment score. “dn/ds” is the ratio of synonymous to non-synonymous changes between the query virus and the HTS found. “Pfam” shows protein family matches for the sequence. “PCR” shows if the sequence was tested by PCR.
(XLSX)
