# Supplementary figures and images for: Accidental Genetic Engineers: Horizontal Sequence Transfer from Parasitoid Wasps to Their Lepidopteran Hosts
Source: PLoS One. 2014 Oct 8;9(10):e109446. doi: 10.1371/journal.pone.0109446 (PMC4190172; doi:10.1371/journal.pone.0109446)

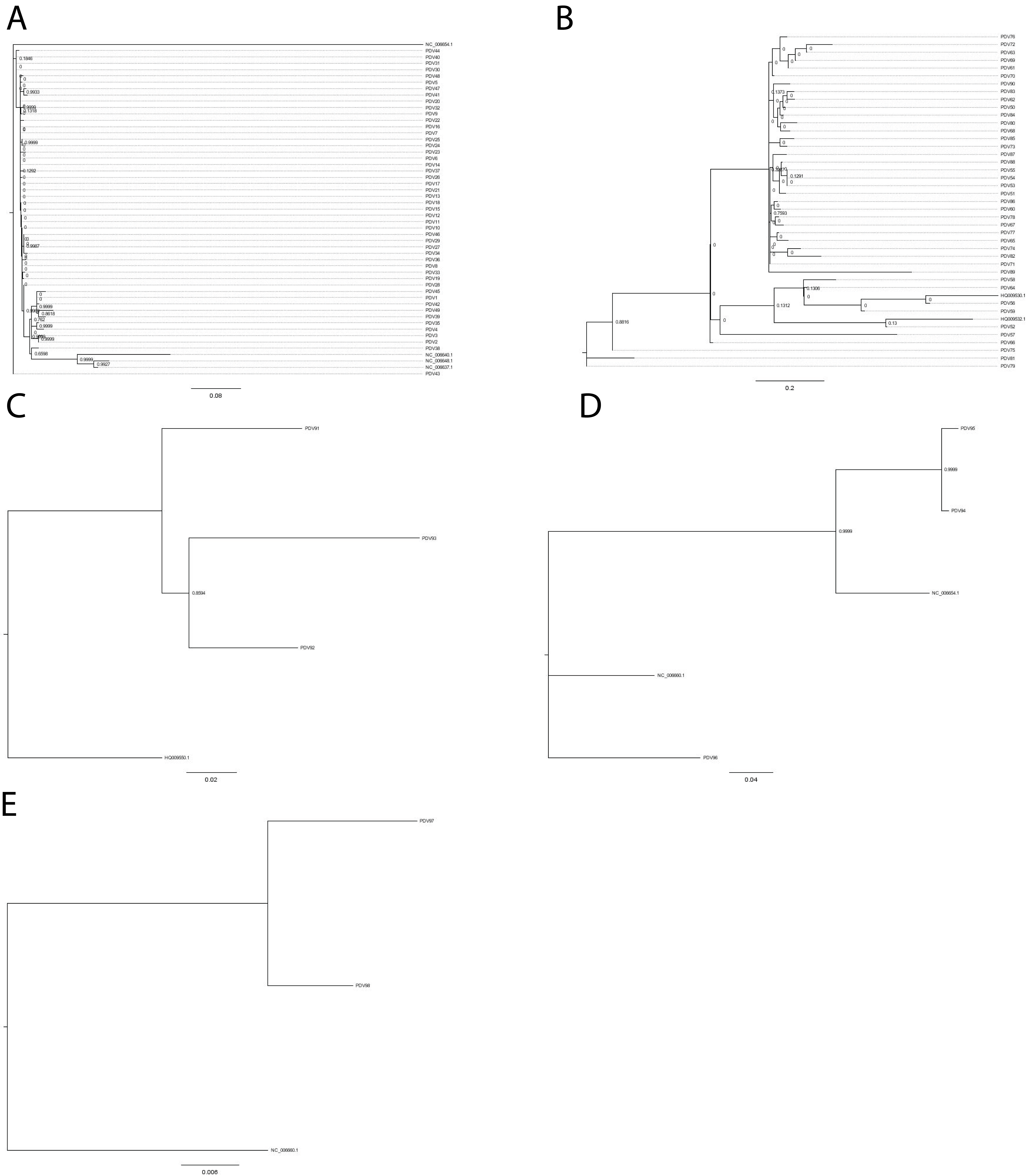

Supplement: Figure S1 — DNA trees for homology groups containing more than one member. Each tree includes all members of the homology group and the original PDV sequences that the members of the homology group matched to. Alignments were performed using DIALIGN in “genomic DNA” mode and trees were created using PHYML A) Homology group 1 B) Homology group2 C) Homology group 3 D) Homology group 4 E) Homology group 5. (PNG) [file pone.0109446.s001.png]

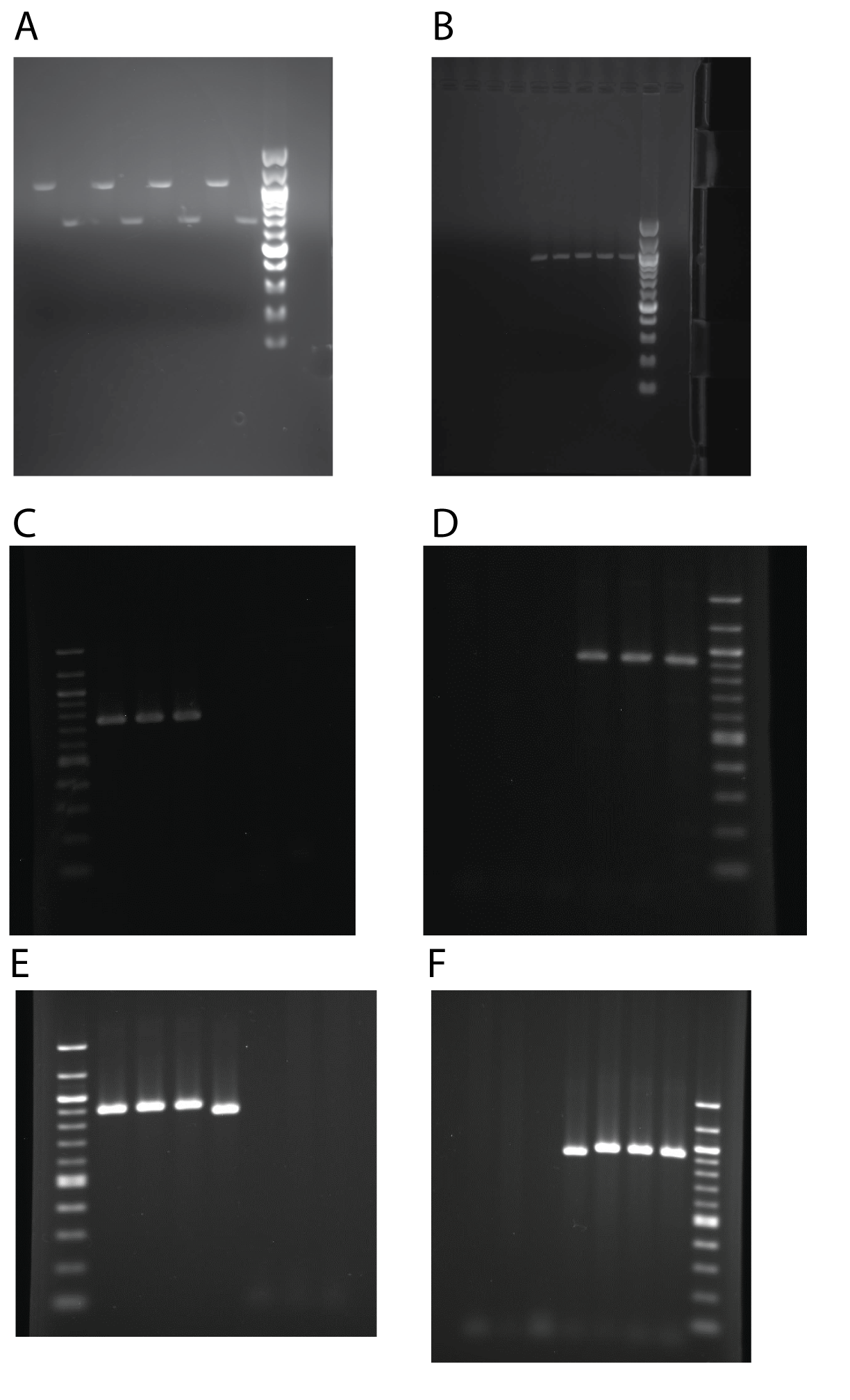

Supplement: Figure S2 — Gels displaying PCR amplification of HTS in Bombyx mori . Figures S2A and S2B show alternative PCR primers amplifying the same regions as the primers used in Fig 4. Figures S2C and S2D show result for primers targeting PDV100. Figures S2E and S2F show result for primers targeting PDV99 Note that in parts C and D the Bombyx strain 106 appears to have a deletion polymorphism yielding a smaller fragment than other strains. Gels were ethidium bromide stained and run with a 100 bp ladder (brighter bands at 500 bp and 1000 bp). A) PCR results for reaction targeting PDV 101 with alternative primers. Lanes alternate between the two different forward primers for the reaction (expected product sizes of 1049 and 686). Tested four strains with each pair of primer sets: 418 (Chinese), 214(Japanese), Nistari (Indian multivoltene), 555(European). B) PCR results for alternative primers targeting PDV32 (expected product size of 947). Five strains were tested: 418 (Chinese), 214(Japanese), 401(Chinese BT-resistant), Nistari (Indian multivoltene), 555(European). C) PCR results for primers targeting PDV100. Lane1: B. mori 214(Japanese). Lane2: B. mori 401(Chinese). Lane 3: B. mori 108(Chinese). Lane 4: B. mori 106(Chinese). Lane 5: Drosophila Melanogaster negative control. Lane 6: Apis Melifera negative control. Lane 7: Chlosynne lacinia (butterfly) negative control D) PCR results for primers targeting PDV100. Lane 1: Chlosynne lacinia (butterfly) negative control. Lane 2: Apis Melifera (honeybee) negative control. Lane 3: Drosophila Melanogaster negative control. Lane 4: B. mori 106(Chinese). Lane 5: B. mori 108(Chinese). Lane 6: B. mori 401(Chinese). Lane7: B. mori 214(Japanese). E) PCR results for primers targeting PDV99. Lane 1: B. mori 214(Japanese). Lane 2: B. mori 401(Chinese). Lane 3: B. mori 108(Chinese). Lane 4: Apis Melifera (honeybee) negative control. Lane 5: Drosophila Melanogaster negative control. Lane 6: Tenebrio molitor(mealworm) negative control. F) Lane 1: Tenebrio [file pone.0109446.s002.png]
